# PD-L1 Expression in Glioblastoma, the Clinical and Prognostic Significance: A Systematic Literature Review and Meta-Analysis

**DOI:** 10.3389/fonc.2020.01015

**Published:** 2020-06-24

**Authors:** Chengcheng Hao, Gang Chen, Huishan Zhao, Yan Li, Jianxin Chen, Hongmei Zhang, Shan Li, Yuze Zhao, Feng Chen, Wenbin Li, Wen G. Jiang

**Affiliations:** ^1^Department of Oncology, Beijing Shijitan Hospital, Capital Medical University, Beijing, China; ^2^Beijing Qinglian Biotech, Co., Ltd., Beijing, China; ^3^Reproductive Medicine Centre, The Affiliated Yantai Yuhuangding Hospital of Qingdao University, Yantai, China; ^4^Department of Neuro-Oncology, Neurosurgery Center, Beijing Tiantan Hospital, Capital Medical University, Beijing, China; ^5^Cardiff China Medical Research Collaborative, Cardiff University School of Medicine, Cardiff, United Kingdom

**Keywords:** glioblastoma, PD-L1, prognostic, clinicopathological, meta-analysis

## Abstract

**Background:** The clinical and prognostic value of programmed death-ligand 1, PD-L1, in glioblastoma remains controversial. The present study aimed to identify the expression of PD-L1 for its prognostic value in glioblastoma.

**Methods:** A comprehensive literature search was performed using the PubMed and CNKI databases. The overall survival (OS) and disease-free survival (DFS) of GBM was analyzed based on Hazard ratios (HRs) and 95% confidence intervals (CIs). Furthermore, Odds ratios (ORs) and 95% CIs were summarized for clinicopathological parameters. The statistical analysis was using RevMan 5.3 software.

**Results:** The meta-analysis was performed by using total nine studies including 806 patients who had glioblastoma. The pooled results indicated that PD-L1 expression in tumor tissues was significantly related to a poor OS (HR = 1.63, 95%CI: 1.19–2.24, *P* = 0.003, random effects model) with heterogeneity (*I*^2^ = 51%). In subgroup analyses, PD-L1 positivity was significantly associated with a worse OS for patients of American and Asian regions, but not for those of European regions. Moreover, PD-L1 expression implied a trend toward the mutation status of the *IDH1* gene [coding the Isocitrate Dehydrogenase (NADP(+))-1 protein] (HR = 9.92, 95%CI: 1.85–53.08, *P* = 0.007, fixed effects model). However, the prediction overall survival (OS) of the patients showed that PD-L1 expression was independent from other clinicopathological features, such as gender and age.

**Conclusions:** Our analyses indicated that high expression of PD-L1 in glioblastoma tumor tissues is associated with poor survival of patients, and PD-L1 may act as a prognostic predictor and an effective therapeutic target for glioblastoma.

## Introduction

Glioblastoma represents the most commonly seen primary malignant brain tumor in adults, characterized by high aggressive behavior and high recurrence rate (1). Multimodality therapies have been suggested and practiced according to NCCN Guidelines, including surgical resection, radiotherapy with alkylating agents such as temozolomide (TMZ) and adjuvant TMZ chemotherapy. However, the outcomes of the treatment are far from satisfactory with the 5-year overall survival being <10% (2–4).

Differently from many other tumors, molecularly targeted therapies for glioblastoma produced very limited advances in prolonging life expectancies of the patients, reasons at least partly attributable to the poor penetration of the Blood Brain Barrier (BBB) by therapeutic agents or by rapidly developing drug resistance (5, 6). In the recent years, it is increasingly recognized that the central nervous system (CNS) interacting actively with the systemic immune system have offered a new exciting theoretical basis and promising opportunities for brain tumor immunotherapy (7–10).

Tumor cells can display immune evasion to weaken antitumor immunity by activating the so-called immune Checkpoint molecules (ICs) (11). Programmed cell death ligand-1 (PD-L1), a “classic” IC molecule, has the effect on induction of T-cell-mediated immune tolerance in tumor local microenvironment, leading to tumor immune escape and tumor growth stimulation, by combination with programmed cell death-1 (PD-1) located on the surface of activated T cells (12). PD-L1 has been shown to be upregulated in various cancer cells and associated with unfavorable prognosis (13–18). Over the past decade, immunotherapies targeting PD-1/PD-L1 axis have made a series of remarkable breakthroughs in prognosis improvement of hard-to-treat solid tumors (including head and neck squamous cell carcinoma, non-small cell lung cancer, gastric cancer, urothelial cancer, cervical cancer, and melanoma) and have entered in the standard clinical practice (19–24). Recently, the expression of PD-L1 on glioma cells has been documented (25, 26). Researches have increasingly concerned over the prognostic evaluation of PD-L1 in patients with glioblastoma. However, whether PD-L1 expression correlates with prognosis in glioblastoma patients remains controversial. Therefore, we assessed the consistency and magnitude of the prognostic and clinical significance of PD-L1 in glioblastoma patients through a systematic review and meta-analysis.

## Materials and Methods

### Literature Search Strategy

The implementation of this systematic review and meta-analysis followed the guideline of PRISM, the Preferred Reporting Items for Systematic Reviews and Meta-Analyses. We systematically reviewed the literature published in the PubMed and CNKI (China National Knowledge Infrastructure) databases (dated to July 2019). The following key words were adopted: (“glioblastoma” OR “GBM” OR “glioma” OR “brain tumor” OR “brain cancer” OR “cerebral tumor” OR “intracranial tumor”) AND (“CD 274” OR “PD-L1” OR “Programmed Cell Death 1 Ligand 1”) without restrictions on languages, regions and publication types.

### Inclusion and Exclusion Criteria

The study adopted the following inclusion criteria: (1) All patients were diagnosed with glioblastoma by histological examination; (2) Hazard ratio (HR) and 95% confidence intervals (CIs) could be available; or the association between PD-L1 and overall survival (OS) or disease-free survival (DFS) with sufficient data were provided.

The study excluded the following: (1) conference abstracts, case reports, reviews, basic research, clinical trials; (2) studies missing available data.

### Data Extraction and Quality Assessment

Two investigators independently reviewed potentially relevant studies in order to minimize bias. A third reviewer was brought in when there were disagreements. We extracted the following data from the included studies: authors, name of the journal, year of publication and ethnicity, number of enrolled patients, tumor histology, PD-L1 expression level, cut off value, detection area, detection methods, and follow-up.

If only survival curves were available, the data could be extracted from the Kaplan Meier curves. The quality of each retrieved article was assessed independently by two assessors according to the Newcastle-Ottawa Quality Assessment Scale (NOS). A total score of 0–9 was assigned to each included study, and studies with a NOS score≥5 were considered to be of high quality (27).

### Statistical Analysis

The association between PD-L1 expression with OS and DFS of patients with glioblastoma was evaluated according to the HR and 95%CI. Statistical heterogeneity among studies was quantified with the Cochran's Q test and the I^2^ statistic. We used a random-effects model to pool the data when evidence suggested significant heterogeneity (I^2^>50% or *P* < 0.1), while a fixed-effects model was conducted otherwise. Subgroup analyses and sensitivity analyses were attempted to explain the origin of heterogeneities. The potential publication bias was estimated by the Begg's and Egger's tests with significance of *P* < 0.05. Review Manager Version 5.3 (Cochrane Collaboration, Oxford, UK) and STATA 15 were statistical packages used in the study.

## Results

### Characteristics of Included Studies

**Figure 1:**
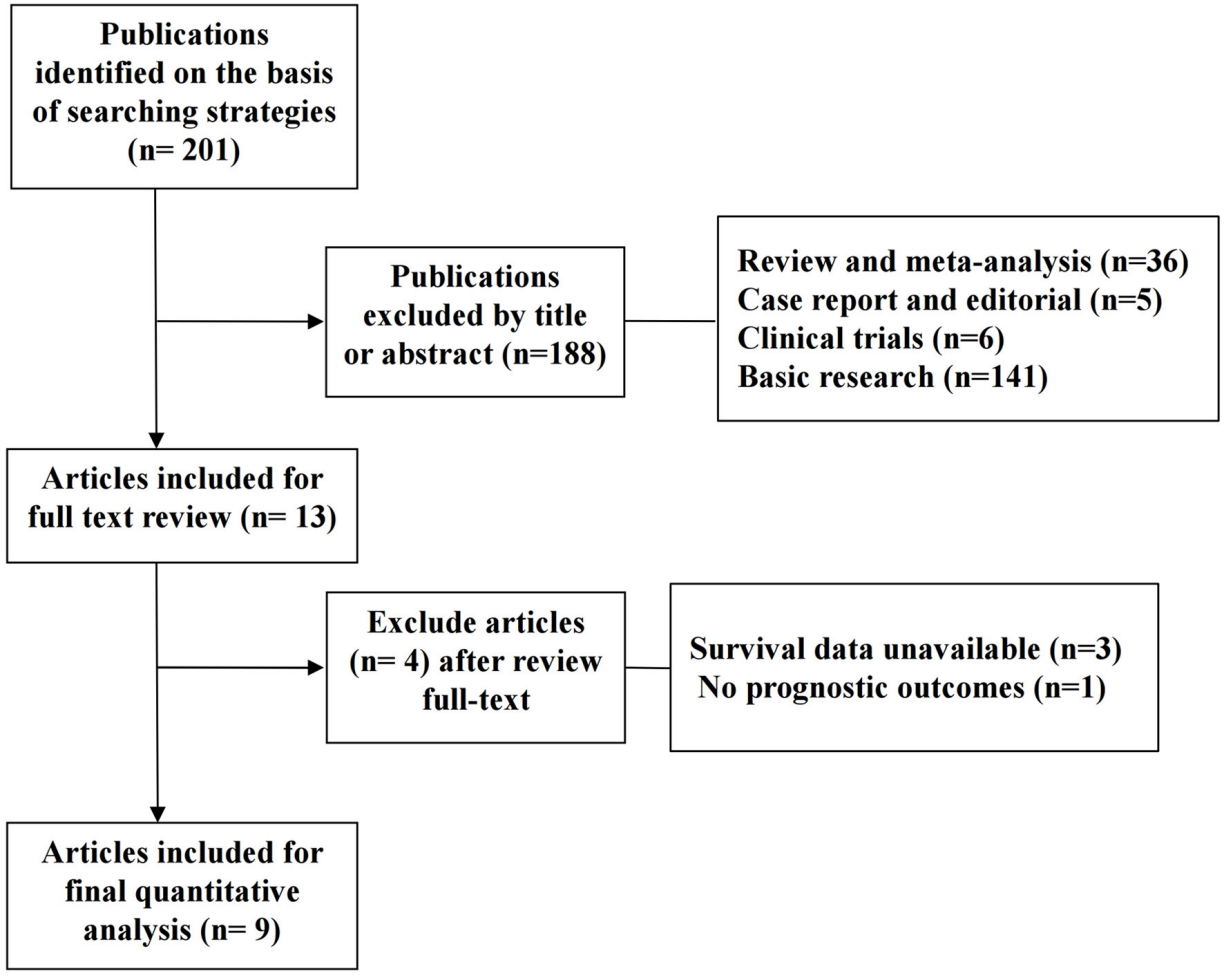
Flow chart of the selection process of studies for inclusion in this meta-analysis.

A total of 201 potentially relevant records were obtained according to the search strategy mentioned above. One hundred and eighty-eight studies were rejected after screening the titles and abstracts. Thirteen studies were included for further evaluation, of which 4 articles without eligible survival data were excluded. Finally, nine studies with 806 patients fulfilled the criteria and entered the meta-analysis. The selection flowchart and the baseline information of the studies are, respectively, displayed in Figure 1 and Table 1.

**Table 1:** General characteristics of included studies.

	**References**	**Journal**	**Year**	**Country**	**No. of patients**	**Treatment**	**Methods**	**Cut-off point (high/low)**	**HR estimation**	**Quality assessment**
1	Liu et al. (28)	The Journal of Neuroscience	2013	Denmark	17	Surgery	IFC	10–100 positive cells	OS	6
2	Berghoff et al. (25)	Neuro-Oncology	2015	Austria	135	Surgery	IHC	5% of positive cells	OS	7
3	Nduom et al. (29)	Neuro-Oncology	2016	USA	94	Surgery	IHC	5% of positive cells	OS	7
4	Zeng et al. (30)	Oncotarget	2016	China	229	Surgery	IHC	5% of positive cells	OS +DFS	8
5	Han et al. (31)	Journal of Pathology and Translational Medicine	2017	Korea	54	Surgery	IHC	5% of positive cells	OS +DFS	8
6	Miyazaki et al. (32)	Journal of Neuro-Oncology	2017	Japan	16	Surgery	IHC	50% of positive cells	OS +DFS	7
7	Lee et al. (33)	Journal of Neuro-Oncology	2017	Korea	115	Surgery	IHC	5% of positive cells	OS	7
8	Pratt et al. (34)	Neurosurgery	2018	USA	125	Surgery	IHC	5% of positive cells	OS	8
9	Hwang et al. (35)	Journal of Neuro-Oncology	2018	South Korea	21	Surgery	IHC	Score≥2 (infrequent small clusters of positive cells)	OS	7

### Association Between PD-L1 Expression and Prognostic Parameters

#### PD-L1 Expression and the Overall Survival (OS) of the Patients

**Figure 2:**
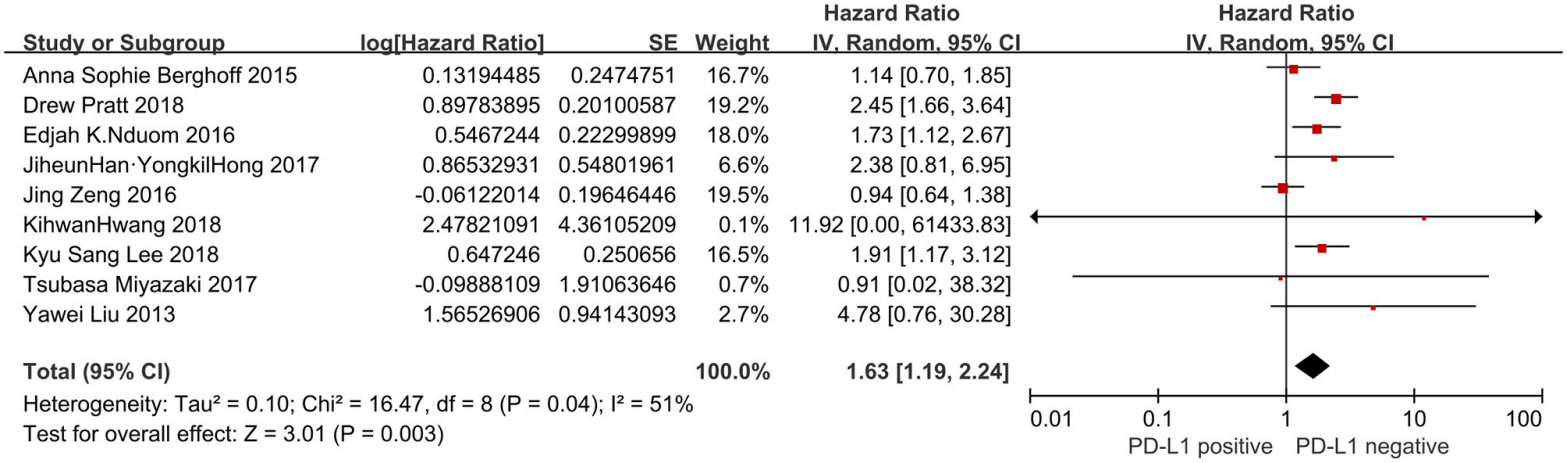
Forest plot of 9 studies evaluating the association between PD-L1 expression and OS in glioblastoma patients.

Nine studies presented OS data (*n* = 806). Significant heterogeneity existed amongst studies included in the analyses (*I*^2^ = 51%, *P* = 0.04). Pooled result by a random-effects model revealed a significantly inverse correlation between PD-L1 overexpression and OS of patients with glioblastoma (HR = 1.63, 95% CI: 1.19–2.24, *P* = 0.003) (Figure 2).

#### PD-L1 Expression and Association With the Diseases Free Survival (DFS)

**Figure 3:**
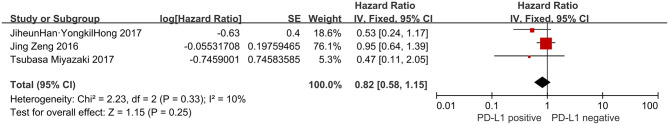
Forest plot of 3 studies evaluating the association between PD-L1 expression and DFS in glioblastoma patients.

As shown in Figure 3, three studies (*n* = 299) focused on DFS and no heterogeneity was existed amongst the studies (*I*^2^= 10%, *P* = 0.33). However, pooled analysis by fixed model did not reveal any significant link between PD-L1 and DFS of patients (HR = 0.82, 95% CI: 0.58–1.15, *P* = 0.25).

### PD-L1 Expression and Clinicopathological Characteristics

#### Age

**Figure 4:**
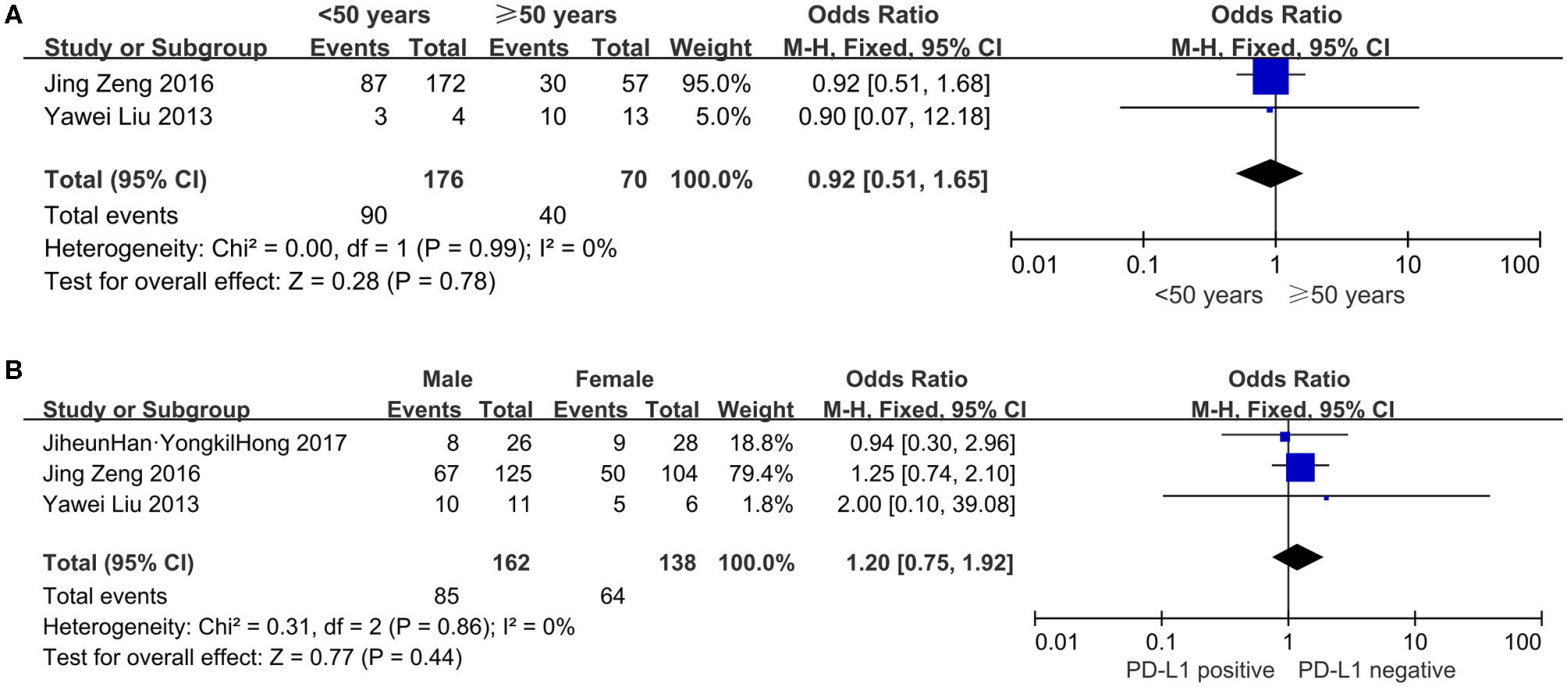
Forest plots for the association of PD-L1 expression with clinicopathological features in glioblastoma patients **(A)** age; **(B)** gender.

Two studies, consisting of 246 patients, assessed the correlation between age and PD-L1 expression. As shown in Figure 4A, 90 (51.14%) of 176 younger patients (whose age defined as younger than 50 yrs) showed PD-L1 expression, compared with 57.14% (40 of 70) of older patients (≥ 50 years of age) who had PD-L1 overexpression. PD-L1 expression did not correlate significantly with age (OR = 0.92, 95% CI: 0.51–1.65, *P* = 0.78).

#### Gender

The dependability between PD-L1 expression and gender was assessed in three studies involving 300 patients. Eighty-five (52.47%) of 162 male patients and 64 (46.38%) of 138 female patients were PD-L1 overexpression. The results indicated that PD-L1 overexpression had no significant association with gender (OR = 1.20, 95% CI: 0.75–1.92, *P* = 0.44; Figure 4B).

#### Ethnicity

**Figure 5:**
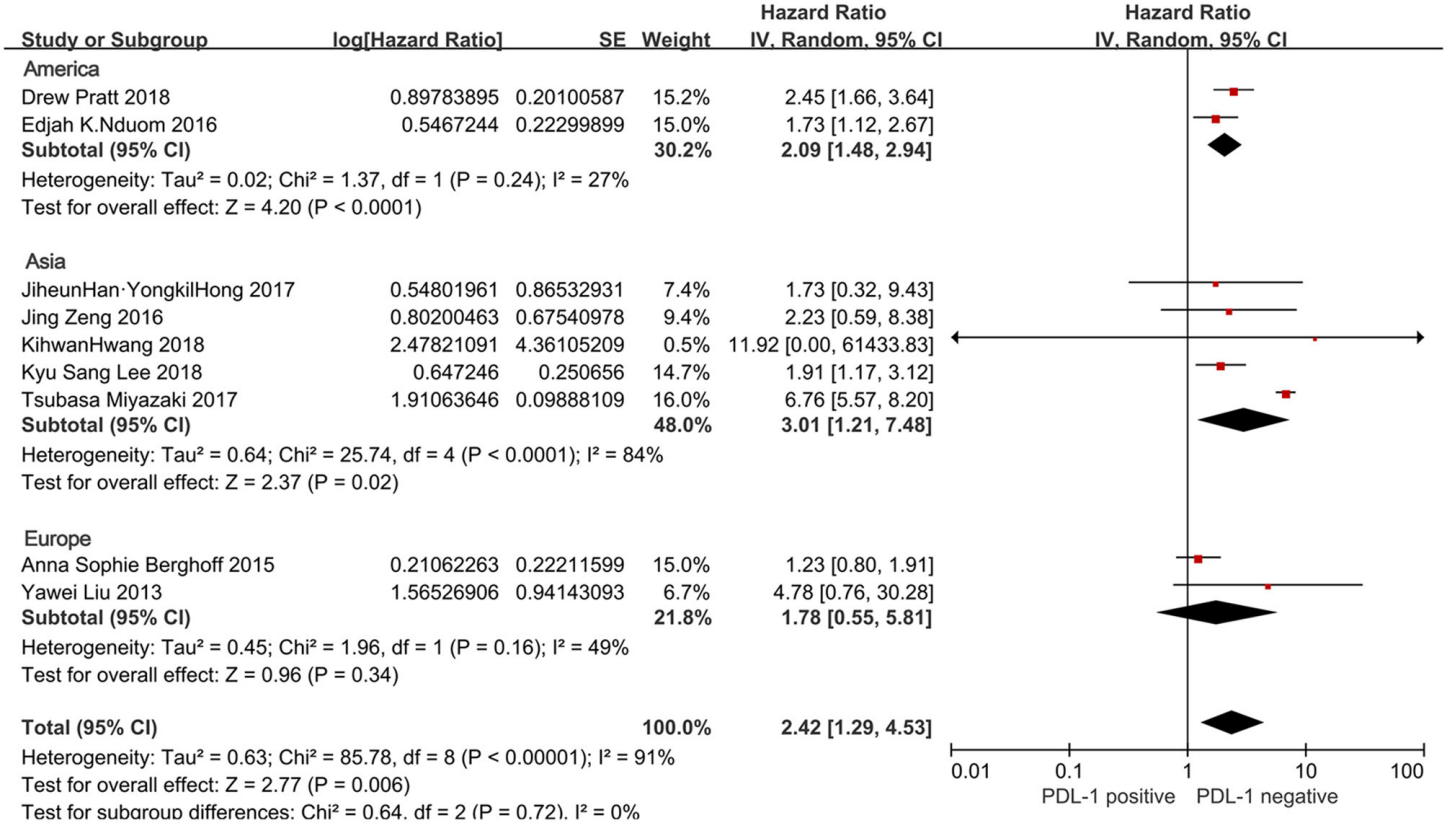
Forest plot for the association of PD-L1 expression with clinicopathological feature of region in glioblastoma patients.

In ethnicity subgroup, the stratified analysis revealed PD-L1 positivity was linked to unfavorable OS in patients from the Asian regions (five studies with 435 cases: HR = 3.01, 95%CI:1.21–7.48, *P* = 0.02) and the American regions (two studies with 219 cases: HR = 2.09, 95% CI:1.48–2.94, *P* < 0.0001), but not in patients from the European studies (two studies with 152 cases: HR = 1.78, 95% CI:0.55–5.81, *P* = 0.34) (Figure 5).

#### IDH1 [Isocitrate Dehydrogenase (NADP(+))-1 Coding Gene] Status

**Figure 6:**
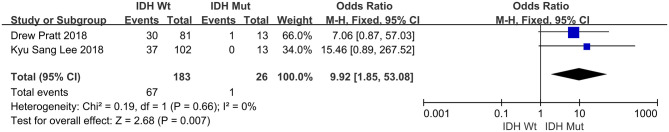
Forest plot for the association of PD-L1 expression with clinicopathological feature of IDH1 status in glioblastoma patients.

Two separate studies, encompassing 209 patients in total, evaluated a possible connection between *IDH1* mutation (namely *IDH1*-wild type vs. with *IDH1* mutation) and PD-L1. Of the 183 tumors which displayed *IDH1*-wild type, 67 (36.61%) were PD-L1 positive. Of the 26 tumors which had *IDH1*-mutantion, one (3.85%) was PD-L1 positive expression. The pooled OR indicated that PD-L1 positivity was closely related to *IDH1* status (OR = 9.92, 95% CI: 1.85–53.08, *P* = 0.007) (Figure 6).

In a subgroup analysis using a random effects model, heterogeneity was revealed in relation to PD-L1 and ethnicity of the patients (*P* < 0.00001, *I*^2^ = 91%).

### Sensitivity Analysis and Publication Bias

**Figure 7:**
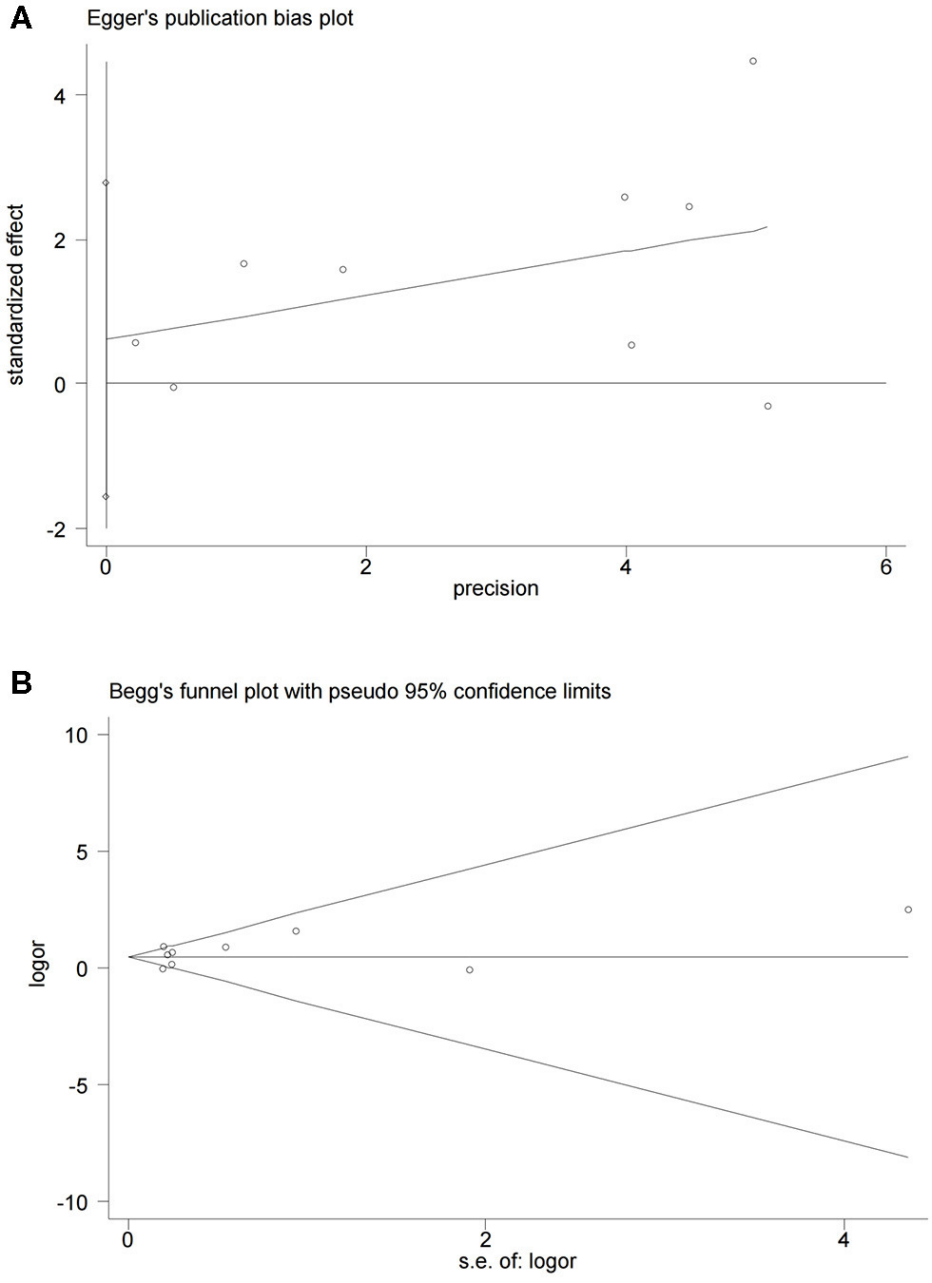
Begg's and Egger's funnel plot with 95% CI for OS publication bias in the included nine studies.

Finally, we found no significant publication bias in the nine studies entered the current analysis, by, respectively, applying the Begg's test and the Egger's test (*P* = 0.917 and *P* = 0.527, respectively) (Figure 7).

## Discussion

PD-L1 is a coinhibitory ligand expressed in many types of tumor cells. It has been indicated that the binding of PD-L1 to its receptor PD-1 induces T cell dysfunction and apoptosis which plays a crucial role in tumor immune evasion. Gliomas have been recognized as an immunosuppressive tumor. The current understanding to glioma-mediated immunosuppression have generated increasing interest in the correlations between PD-L1 expression and survival for gliomas, particularly glioblastomas. However, the published results about glioblastomas remain controversial. In 2013 Liu et al. first reported that the expression of PD-L1 in seventeen patients with glioblastoma is a possible indicator for poor clinical outcome (28). Using level 3 Illumina RNASeq, Nduom et al. also found PD-L1 overexpression was indicative for shorter survival time in 149 patients with glioblastoma from (data from the Cancer Genome Atlas (TCGA) dataset) (29). Various other studies showed similar results (31, 33, 34). However, in a retrospective study of 117 newly diagnosed glioblastomas as well as a TCGA database analysis comprising 446 glioblastoma patients, researchersdid not find a significant connection between PD-L1 and the OS (25). The similar views were also taken in several other analyses (30, 32). To clarify a reasonable evidence-based conclusion, a meta-analysis including 9 studies with a total of 806 patients was performed. The present meta-analysis showed that PD-L1 positive expression was significantly associated with poor OS (HR = 1.63, 95% CI: 1.19–2.24, *P* = 0.003) in glioblastoma patients after surgery; however, there were insufficient evidence to suggest that PD-L1 was related to DFS (HR = 0.82, 95% CI: 0.58–1.15, *P* = 0.25). These results suggested that PD-L1 positive expression might be a negative prognostic factor in glioblastomas.

To further explore the potential sources of heterogeneity in the relationship between PD-L1 and overall survival in glioblastoma, we utilized subgroup analyses. The results confirmed that the significance of PD-L1 in OS was not affected by gender and age, collectively suggesting that this relationship is independent of these factors in tumor type. The influence of PD-L1 on the OS of multiethnic patients was also explored. Patients were classified as from Asia, America and Europe. Different combined HRs and *P*-values for OS were shown in different ethnic groups: PD-L1 overexpression was significantly associated with poor OS for patients from Asia and America, while no significant association for the survival of patients from Europe survival, which suggested that racial differences may be a potential origin of heterogeneity in glioblastoma. This “ethnic biasedness” of PD-L1 has been observed in several clinical studies for patients with certain other types of solid tumors. KEYNOTE-181, a phase 3 trial of Pembrolizumab (P) vs. chemotherapy (paclitaxel, docetaxel or irinotecan) in patients with esophageal squamous cell carcinoma, showed that P was superior to chemotherapy for OS in patients with PD-L1 positive expression (CPS≥10) in the global cohort, especially in the Chinese subgroup (36). Similarly, in the KEYNOTE-062 (a study of Pembrolizumab vs. chemotherapy in patients who had advanced gastric or gastro-esophageal junction cancer), P didn't bring significant survival benefits as the first-line treatment for PD-L1-positive (CPS≥1) population in the full global cohort (37). However, in further ethnicity subgroup analysis, P showed lower risk of death in Asians with PD-L1 positive expression (CPS≥1) when compared to chemotherapy, but not in Europeans, Americans and Australians. The findings in stratified analyses revealed that PD-L1 holding a prognostic role in different ethnic groups may have potential implications inimmunotherapy and prognostication for stratify patients. It is possible that the immunogenetics might to some extent differ in different races (38). So the subgroup analysis stratified by ethnicity may be necessary for accurately assessing the potential prognostic value of PD-L1 and the efficacy of relevant immunotherapy drugs in further clinical studies for glioblastoma.

According to the updated 2016 World Health Organization (WHO) classification of diffuse gliomas, Isocitrate dehydrogenase (*IDH*) has been considered as one of the important molecular biomarkers that has diagnostic, prognostic and predictive application (39–42). Studies have reported that glioblastomas which are hotspot mutation in *IDH1* (an isoform of *IDH*) generally have a significantly better prognosis compared with *IDH1*-wildtype glioblastomas (43–45). Moreover, the expression of PD-L1 in diffuse gliomas might be directly influenced by *IDH1* mutational status (33, 46, 47). Therefore, the relation between PD-L1 expression and *IDH1* status was further investigated in the stratified analysis. We found that *IDH1*-wildtype status in glioblastoma was PD-L1 expression positive. The result confirmed recent findings of a PD-L1/*IDH1*-wildtype association. The hypothesis about its mechanism of this connection was that *IDH1* mutation can result in the PD-L1 promoter hypermethylation that downregulates the expression of PD-L1 (48, 49). So the PD-L1 immune checkpoint inhibitors might not be advisable because of the low PD-L1 expression in patients with *IDH1*-mutant glioblastomas.

Recent research has showed that tumor cells in gliomas can regulate PD-L1 expression via two major mechanisms to mediate immune evasion, “adaptive resistance” mechanism and “innate resistance” mechanism (50). The former is for extrinsic induction of PD-L1. IFN-γ, a proinflammatory cytokine primarily produced by tumor infiltrating lymphocytes (TILs), can induce PD-L1 upregulation via NF-κB /PKD2 signal pathway (51–53). The latter is proved to mediate intrinsic induction of PD-L1. When there is a lack of TILs, PD-L1 induction in glioma cells might depend on multiple oncogenic signaling pathways (such as JAK/STAT 3, PI3K/Akt/mTOR/S6K1 and EGFR/MAPK pathway) or oncogene mutations (such as ALK, EGFR and PTEN) (11, 54–60).

It has been indicated that PD-L1 may be a valuable therapeutic target in cancer immunotherapy (9, 61). Recently, promising preclinical data in murine models of glioma have provided support for PD-L1 checkpoint inhibitors implementation in GBM patients (62–64). However, early clinical trials on the effectiveness of PD-L1 blockade agents are still limited and elusive. A combination between Anti-PD-L1 mAb durvalumab and bevacizumab is now being tested in a phase 2 open label, non-randomized clinical trial for GBM (cohort B, NCT02336165) (65). Interim results of durvalumab monotherapy revealed low SAE (severe adverse events) rate of 10% and efficacy with OS-6 of 42% and PFS-6 (progression-free survival) of 20%. Trials for other cohorts (cohort A: newly diagnosed MGMT-promoter unmethylated GBM, cohort C: refractory recurrent GBM) are still ongoing. Atezolizumab, a humanized antibody to PD-L1 that has been approved for second-line treatment for patients with advanced or metastatic NSCLC (non-small cell lung cancer) and urothelial cancer, is also being studied in a phase 1a clinical trial for multiple solid tumors, including GBM (PCD4989g; NCT01375842) (66). Results showed that Atezolizumab was safe and well-tolerated in patients with GBM. Glioblastoma was considered a type of “immunologically cold tumor” due to the relative lack of tumor infiltrating T cells in the tumor micro-environment (TME) and high selectivity of BBB (67–69). The “cold” phenotype of GBM may limit immunotherapy efficacy. Combinatorial treatment approaches targeting immune-suppression or BBB permeability may help shift the “cold” microenvironment and enhance response to immune checkpoint blockade in GBM, including radiation therapy, chemotherapy, other immunotherapies (e.g., Chimeric antigen receptor T-cells, oncolytic virus), and bevacizumab (70, 71).

So far as we are aware, the present study is the first meta-analysis to systematically estimate the correlation between PD-L1 and clinical outcomes and clinicopathological factors in glioblastoma. While some limitations need attention. Firstly, different analysis strategy of IHC and inconsistent cut-off values of PD-L1 expression may lead to heterogeneity between studies. Thus, a standardized approach for protein expression should be set up to improve consistency and veracity in the measurement of PD-L1 for future studies. Secondly, some subgroups, such as the *IDH1* status group, had small sample sizes. Thirdly, the investigation about the correlations between PD-L1 and clinical features, including as tumor size, tumor site and surgical approach are not performed due to the deficiency of related original information.

In conclusion, our meta-analysis shows that high PD-L1 expression is strongly correlated with unfavorable prognosis for GBM patients. PD-L1 may present itself as a valuable target for immunotherapy in clinical practice. Additional high-quality, larger-scale prospective studies are needed to provide validate the potential value of PD-L1 for the prognosis and treatment of GBM patients in the future.

## Data Availability Statement

The datasets generated for this study are available on request to the corresponding author.

## Author Contributions

CH and GC designed this study, searched the literature, screened identified studies and extracted data, and wrote the manuscript. Disagreements were resolved by discussion with WJ and WL. HZhao, FC, YL, SL, HZhang, YZ, and JC assisted in the literature search and screening. WJ and WL assisted in the designing of the study and the data analyses, and supervised the study. All authors have read and approved the final version of this manuscript.

## Conflict of Interest

GC was employed by the company Beijing Qinglian Biotech, Co., Ltd., Beijing, China. The remaining authors declare that the research was conducted in the absence of any commercial or financial relationships that could be construed as a potential conflict of interest.
